# Bier's Method and Ulcers of the Leg

**Published:** 1908-04-04

**Authors:** 


### Points in Treatment.

### BIER'S METHOD AND ULCERS OF THE LEG.

There is probably no common and non-fatal affection that is more troublesome to treat successfully than non-specific ulceration of the leg. The skin and subcutaneous tissues in the inner aspect of the lower third of the leg, where these ulcers are commonest, are comparatively poor in their blood supply; the part is particularly liable to small injuries; and when once an ulcer has formed, its surroundings very quickly become indurated and hide-bound from capillary lymphatic obstruction. There may or may not be varicose veins in the leg at the same time; whether such varicosity exists or not, the ulcers are commonly termed "varicose" when it is desired to distinguish them from syphilitic ulcers; probably a better adjective than varicose would be "malnutritional."

It is the malnutrition of the part which makes it so difficult to cure the ulcers. Anything which assists in promoting the better nutrition of the leg also helps the ulcer to heal. One of the simplest measures is to keep the leg raised so that the foot is on a level with the heart; the blood is then able to return by the veins with greater rapidity than it can when the leg is down; and if the patient can afford to lie up in bed for a good many weeks, or even months, there are few of these ulcers that cannot be got well, at least for the time being.

The difficulty is that the very class of persons that is most liable to malnutritional ulceration of the legs is also the class that is obliged to be up and about, seeing to household and other work. Even when a certain length of time can be spared for continuous rest in bed the patient is eager all the while to be up and about again at the earliest opportunity. The result of this is that, no sooner is the ulcer nicely skinned over again with fresh epidermis than the patient, having been in bed for six weeks or two months, perhaps, deems herself well, and the leg is used again too soon. The new-formed epidermis has not had time to become mature; and the slightest injury or excoriation causes it to ulcerate afresh; nay, even without injury, the dependent position of the leg by itself may cause recurrence of the ulceration owing to return of the original state of malnutrition in the part from stagnation in the dependent veins.

The crux of the whole question is the length of time that can be devoted to the resting of the ulcerated leg up in bed, and anything which can bring the cure to a more advanced stage of maturity in a given space of time is bound to be a boon in the treatment of the condition.

We do not propose to deal with the ointments or fomentations or caustics that may be applied to the surface of the ulcers themselves; nor with the remedies such as calcium chloride or calcium lactate, potassium iodide, colchicum, and so forth that may be given internally. These we have already discussed elsewhere. It is our purpose to discuss to-day a simple procedure by which the rate of repair in the ulcer may be considerably accelerated during the period of rest, so that the length of time during which the patient must lie with the leg up can be considerably curtailed. This simple procedure is known as Bier's passive hyperæmia method.

The process is very easy, and it requires no special apparatus beyond an elastic bandage which is used in a way very similar to a tourniquet. A Martin's bandage is very convenient for the purpose, and it should be about three inches wide. This is applied round the leg, proximal to the ulcer and as remote from it as may be convenient. The affected region of skin should on no account be subjected to pressure; a good place for the bandage is just above the knee.

The most important point about the bandaging is the tightness with which it is done; and no absolute rule can be laid down, for one patient can bear it much tighter than another can. Broadly speaking, the bandaging has to be such that the return flow of blood has only to be impeded, not stopped, whilst the temperature of the distal end of the limb must not be lowered. The tightness of the bandage must be considerable, but yet something less than is required when, for example, venesection is to be performed. The parts beyond the bandage will swell, and become reddened; if the application is too tight the colour may become bluish or even white, in which case the bandage should at once be loosened. There is a subjective feeling of tension in the parts in and around the ulcer, and the patient's sensations afford a very good clinical guide as to how tight the bandage should be. That which is most comfortable to the patient is approximately the best for the treatment, which should stop short of any painfulness at the site of the bandaging or of acute discomfort in the parts beyond.

The length of time the bandage should be left on will vary in different cases; some prefer continuous application for many hours, others periodic bandaging for short periods at a time. Probably the latter is the more convenient and comfortable in most cases, the bandage being applied for an hour at a time night and morning. There is no absolute need for the patients to remain in bed, at least in so far as the Bier's treatment itself is concerned, and the procedure has been successfully used in out-patients who, after having the bandage on for an hour under inspection, to see that all is well, have been allowed to go home with it *in situ*, to be left on till next day. This, however, applies to the treatment of inflammatory conditions such as whitlows and the like rather than to ulcers of the leg; for the latter, rest in bed is the ideal condition, with Bier's treatment to accelerate the process of repair.

Precisely how the treatment acts is not known. Theoretically, if it is a bad thing for the ulcerated leg to be dependent it should also be the reverse of good to produce an artificial venous stasis in the part. Practical experience shows this not to be the case, however; and the intermittent passive hyperæmia induced by the bandaging undoubtedly does a great deal of good in these cases. The procedure is very simple, and it costs very little. There can be no doubt of its value as an additional means of treating what is otherwise a very refractory disease.

